# Profiling *Mycobacterium ulcerans*: sporulation, survival strategy and response to environmental factors

**DOI:** 10.2144/fsoa-2022-0044

**Published:** 2023-03-23

**Authors:** Eunice Ampadubea Ayerakwa, Molly Kukua Abban, Abiola Isawumi, Lydia Mosi

**Affiliations:** 1 West African Centre for Cell Biology of Infectious Pathogens, LG 54 Volta Road, University of Ghana, Legon Accra, Ghana; 2 Department of Biochemistry, Cell & Molecular Biology, LG 54 Volta Road, Legon Accra, University of Ghana

**Keywords:** Buruli ulcer, environmental pathogen, mycobacteria, *Mycobacteria ulcerans*, sporulation, stress response mechanisms

## Abstract

*Mycobacterium ulcerans* is the causative agent of Buruli ulcer – a necrotizing skin infection. As an environmental pathogen, it has developed stress response mechanisms for survival. Similar to endospore formation in *M. marinum*, it is likely that *M. ulcerans* employs sporulation mechanisms for its survival and transmission. In this review, we modeled possible transmission routes and patterns of *M. ulcerans* from the environment to its host. We provided insights into the evolution of *M. ulcerans* and its genomic profiles. We discuss reservoirs of *M. ulcerans* as an environmental pathogen and its environmental survival. We comprehensively discuss sporulation as a possible stress response mechanism and modelled endospore formation in *M. ulcerans*. At last, we highlighted sporulation associated markers, which upon expression trigger endospore formation.

## Plain language summary

Buruli ulcer is an infectious disease characterized by extensive sores on the skin and soft body tissues. The disease is caused by a bacterium called *Mycobacterium ulcerans* and is mainly found in tropical countries. Over the years, several attempts to understand the means by which humans get into contact with this bug as well as how it thrives in its host remain futile. In this review, we describe a possible survival strategy, known as sporulation, that is adopted by the pathogen for dispersal and survival.

*Mycobacterium ulcerans* is the causative agent of Buruli ulcer (BU) and it is an environmental mycobacterium usually found in an aquatic and swampy environment. These environments expose the pathogen to harsh conditions such as extreme temperature, nutrient deprivation and harsh chemicals [1]. As such, the pathogen may develop stress response mechanisms to survive these harsh conditions. The Formation of dormant spores is a major stress response mechanism for most environmental pathogens including some species of *Bacillus, Clostridium* and *Mycobacterium*. While this kind of spore forming mechanism allows the pathogen to survive under stressed conditions, it may also help with dispersal to new environments [2]. Recently, sporulation has been a topic of interest among members of the mycobacteria genus such as *M. marinum*, *Mycobacterium bovis* and *M. avium* subsp. *paratuberculosis* [3]. However, little has been shown in *M. ulcerans*, although the identification of its route for transmission is still unclear. Therefore, reviewing its spore-forming potential as a survival mechanism and possible means of disease transmission is essential to public health.

## Buruli ulcer

**Figure 1.**
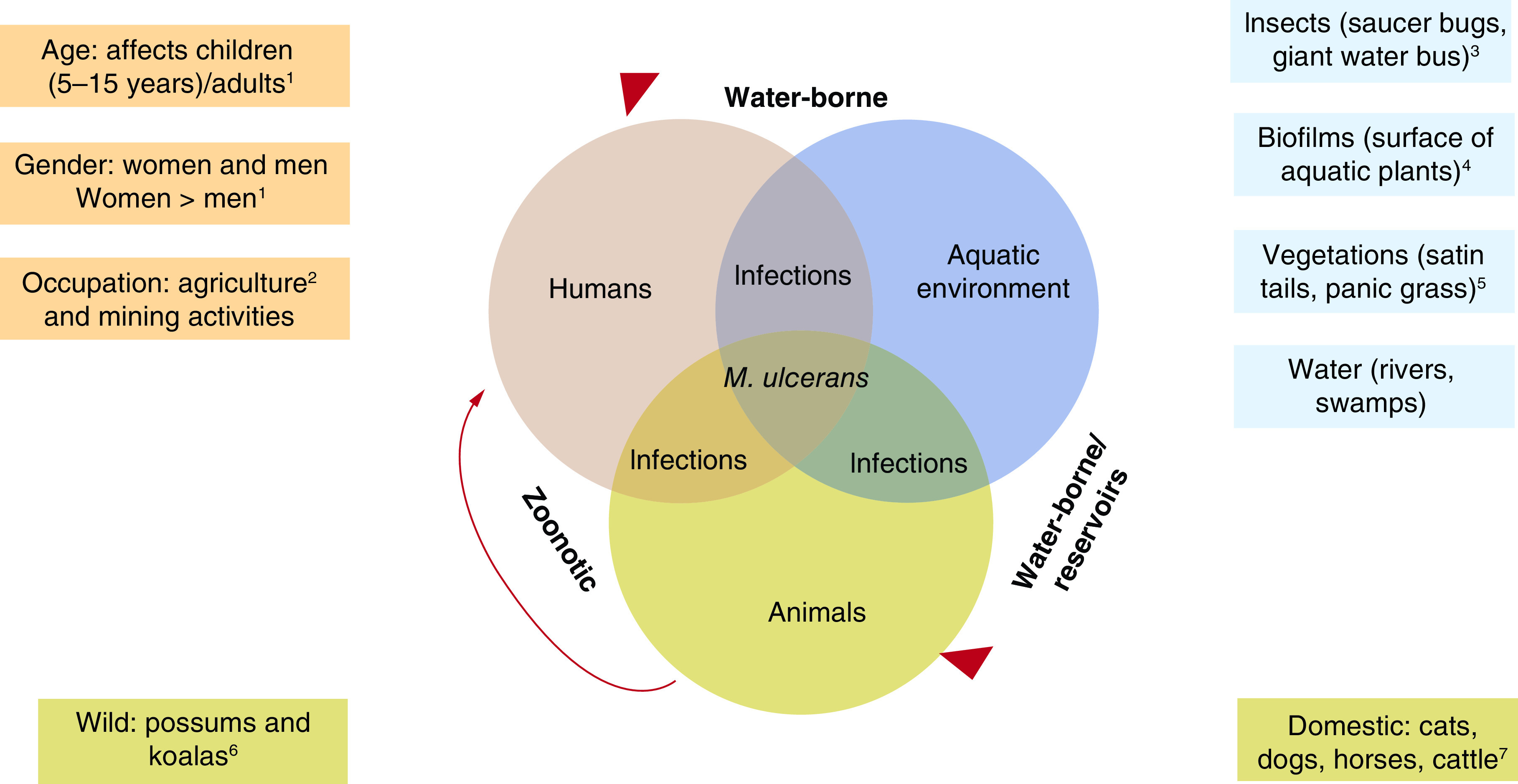
Proposed transmission patterns of *Mycobacterium ulcerans* showing the role of the aquatic ecosystems and animals in the transmission of *M. ulcerans* from the environment to humans. (Figure designed by authors: ^1^[5], ^2^[7], ^3^[13], ^4^[38], ^5^[72], ^6^[11] and ^7^[15]).

BU is a neglected tropical disease characterized by progressive damage to the skin and soft tissues [4]. The disease has been reported in countries found in America, Africa, Asia and Western Pacific and it affects people of all ages. However, children below 15 years are the most affected [5], and lesions usually appear on the lower limbs [6]. BU is the third most common mycobacterial infection after leprosy and tuberculosis; however, identification of a definite mode of transmission is a major public health concern. Unlike TB and leprosy, human-to-human spread of BU is rare. Hypothetically, *M. ulcerans* is acquired from the environment and individuals living in remote areas near aquatic environments are usually at higher risk of developing infections [7]. From these environments *M. ulcerans* can gain entry into the human host through skin cuts or wounds [8]. Also, BU transmission has been associated with insect bites with mosquitoes as possible reservoirs or vectors [9]. The possibility of transmission through aerosols has been proposed [10]. Aquatic insects could also serve as reservoirs for *M. ulcerans* [11]. Water bugs and insects that belong to the Naucoridae and Belostimadae families are considered reservoirs and possible transmission vectors (Figure 1 [12,13]).

BU usually starts as a painless nodule or papule that progresses into severe skin ulceration with undermined edges [14]. Typically, these ulcers are associated with severe coagulative necrosis [15]. Mycolactone, a lipid-like plasmid encoded toxin secreted by *M. ulcerans* is the major virulence factor that mediates pathogenesis. Unlike other *Mycobacterial* spp., *M. ulcerans* exists as an extracellular cluster of bacilli that usually concentrates at the center of the necrotic areas [16]. *M. ulcerans* could exist in macrophages within inflammatory filtrates, suggesting that the pathogen can be intracellular and extracellular. The possibility of mycolactone inhibiting *M. ulcerans* uptake by macrophages is high [16]. The determinants of the pathogenesis of *M. ulcerans* includes ability to behave as an intracellular pathogen and synthesize mycolactone [17]. Mycolactone diffuses through surrounding tissues exerting multiple effects (cytotoxicity and immunosuppression) on cells; although its mechanism is less understood, several targets of this toxin have been identified. Purified vesicles extracted from *M. ulcerans* extracellular matrix were highly toxic, which shows that mycolactone is secreted and stored in the extracellular matrix [16]. As a result of mycolactone secretion, host cells cytoskeleton and tissue structure are disrupted, leading to cell death. Mycolactone also targets the Wiskott–Aldrich syndrome proteins (WASP) and neural WASP, which controls actin dynamics in adherent cells [18]. Ulceration and an eventual cell loss result from induced apoptosis caused by inhibition of protein translocation into the endoplasmic reticulum and cell detachment [19].

### Mycobacterium ulcerans

*M. ulcerans* has evolved diverse mechanisms including highly resistant cell envelope to dehydration [20], and the synthesis of photoprotective compounds [21], to survive under harsh environmental conditions. Also, a waxy cell envelope rich in mycolic acids and lipids, conferring the ability to resist decolorization with acidic organic solvents. *M. ulcerans* is a slow growing nontuberculous mycobacterium which mostly affects humans. It grows at 29–33°C under microaerophilic conditions (5% CO_2_) [22]. Although *M. ulcerans* is an environmental mycobacterium, attempts to isolate pure cultures from their supposed habitats have been unsuccessful and difficult; however, it has been isolated from aquatic insects and biofilms [23]. In the human host, *M. ulcerans* extracellularly produce macrolide toxin encoded by a plasmid (174 kb), increasing its virulence and enabling it to establish severe BU infections. *M. ulcerans* is thought to have evolved from *M. marinum* by acquiring this virulence conferring plasmid. *M. marinum* is an atypical mycobacterium that causes infections in fishes and humans. Human infections may occur following exposure to aquatic environments and animals (fish and aquatic bugs). However, *M. marinum* infections are minor and are characterized by intracellular bacteria and granulomatous lesions [24].

### Genome structure & evolution of *M. ulcerans*

*M. ulcerans* and *M. marinum* share 98% similar genome and genetic markers [25]; however, the differences are as a result of gene insertions or deletions. Analysis of the 3′ end of the highly conserved 16S rRNA sequence of mycobacteria shows that *M. ulcerans* differs from *M. marinum* at just one nucleotide position or single nucleotide polymorphism [26]. *The M. ulcerans* strain Agy99 isolated in Ghana, was the first genome sequenced [26]. There were two replicons, 631,606 bp chromosome and the 174,155 bp virulence plasmid pMUM001 with the genome. The chromosome consists of about 4160 protein coding genes and 771 pseudogenes. The genome of *M. ulcerans* has a high G + C content (65% chromosomal and 62.5% plasmid genome) with 12 insertion elements, including the well characterized *IS2404* and *IS2606* insertion sequence [27]. These IS*2404* is present in high copy numbers in the *M. ulcerans* core and plasmid genome, however absent in *M. marinum* [28]. The insertion sequences are distributed within the genome, and are sometimes inserted in the middle of some genes. Insertion of these sequences in the middle of genes interrupts coding sequences and inhibits their expression. The genes that are interrupted by insertion sequences become inactivated and are likely to form pseudogenes [29]. In the *M. ulcerans* genome, there are about 771 pseudogenes, and this may be because *M. ulcerans* has a higher number of insertion sequences that are constantly inserted into functional genes [29]. An example of gene interruption and pseudogenization is observed in the *otsB1* and *otsB2* genes present in both *M. tuberculosis* and *M. ulcerans*, which are essential for cell wall biogenesis. However, in the *M. ulcerans* genome, the *otsB1* gene has been disrupted by an *IS2404* and has lost its function [30]. Genomic DNA deletion, loss of gene function and pseudogene formation are features of genome reduction or downsizing in the *M. ulcerans* [31]. Some pathogens that undergo genomic reduction adapt easily to a foreign host and harsh environment [30]. Consequently, genes that are not very essential for their survival in this environment or host become functionally silent and redundant. This suggests that *M. ulcerans* is capable of undergoing genetic alterations to adapt to a particular ecological niche [30].

*The M. ulcerans* genome also contains two prophages that are structurally similar to other mycobacteriophages [29]. These are the 18 kb *phiMU01* and the 24 kb *phiMU02* with an 18 and a 17 coding sequences (CDs). Comparative genomic analysis of the *M. ulcerans* genome has also shown the deletion or absence of some genes that encode immunogenic proteins. For example, the *esxB-esxA* gene clusters are highly immunogenic mycobacterial proteins essential for host’s immune response [32]. The *M. marinum* genome contains two copies of these *esxB-esxA* cassettes, but several *M. ulcerans* strains have only one copy of this gene, and others have none. This evolutionary feature can enhance the immune evasion of the pathogen in its host. The hallmark of *M. ulcerans* evolution is the acquisition of a virulent plasmid *pMUM001*, which differentiates the pathogen from *M. marinum*. It harbors three essential genes: *mlsA1, mlsA2* and *mlsB*, encoding polyketide synthases required for mycolactone synthesis [33]. Six structural types of mycolactone have been characterized from the various mycolactone producing mycobacteriums (A/B, C, D, E, F and G^2^). Other mycolactone producing mycobacterium, including *M. shinshuense*, *M. pseudoshottsii* and *M. ‘liflandii*,’ (which may not be necessarily associated with BU) have been described with a common *M. marinum* progenitor [34]. They have very similar phenotypic and genotypic characteristics to *M. ulcerans* and have been argued not to be classified as separate species [34]. Overall, the main events in *M. ulcerans* evolution include DNA rearrangements, gene deletions, a proliferation of Insertion sequences and the acquisition of plasmid, which are characteristic of bacteria that have undergone a bottleneck evolution. These features, however, enable the pathogen to adapt to a specific environment or host, escape immune defence and contribute immensely to its pathogenicity [30].

### Reservoirs of *M. ulcerans* as an environmental pathogen

The genome or genetic diversity of *M. ulcerans* supports its adaptation and survival under different environments; hence, it could be described as an environment pathogen [26]. *M. ulcerans* DNA has been isolated from several swampy environments which further establishes its environmental habitat. Molecular techniques including DNA sequencing and PCR amplification of the *KR-B* gene (mycolactone ketoreductase-B gene) and IS*2404* sequence showed that *M. ulcerans* are present in water filtrates, detritus and plants in some endemic and nonendemic regions of Ghana and Ivory Coast [35,36]. The isolation of *M. ulcerans* from water bodies in BU endemic regions has made it important to consider water as a major reservoir for the pathogen. Also, aquatic plants provide suitable habitat for *M. ulcerans* [37]. *M. ulcerans* can possibly be transmitted through aquatic vegetations such as *Imperata* sp. (satin tails) and *Panicum* sp. (panic grass) [37]. The pathogen has been reported to form biofilms on the surfaces of these aquatic vegetations [38]. Marsollier *et al.* further showed that aquatic plants stimulate the growth and biofilm formation of *M. ulcerans* by addition of a crude extract from green algae to BACTEC 7H12B culture medium [39].

Aquatic environments indicating the presence of *M. ulcerans* had different plant communities, suggesting that the pathogen’s dispersal is unrestricted. The concept of *M. ulcerans* biofilm formation is unclear; however, Marsollier and his colleagues subsequently demonstrated that it adopts a biofilm like structure *in vivo* and *in vitro* with different biofilm layers or components different from other bacterial biofilms [39]. These biofilms have a high amount of extracellular matrix, which contains vesicles shown to be reservoirs of mycolactone [40]. Aquatic insects such as creeping water bugs and giant water bugs have also been implicated in BU disease transmission. The ability of some infected aquatic insects to transmit *M. ulcerans* to laboratory mice through bites has also been demonstrated [11], however, the insect transmission hypothesis is mostly mechanical and not biological. Also, some aquatic insects (Naucoridae, Belostomatidae, Nepidae and Heteroptera) can serve as transient hosts of the pathogen before transmission to their respective host [41]. Aquatic heteropterans could contaminate water and can further infect people away from their sources as they fly around. Animals including wildlife (possums, rodents, shrews, koalas), livestock (goats), pets (dogs) and recreational (horses) are possible reservoirs of *M. ulcerans* (zoonotic transmission) (Figure 1) [42,43]. In the endemic region of Australia, 43% of ringtail possums and 29% of brushtail possums were positive for *M. ulcerans* and 1% for possum faecal samples in nonendemic regions [44]. These indicated terrestrial mammals as possible reservoirs of *M. ulcerans*. In Ghana, however, an attempt to isolate *M. ulcerans* from rodents and shrews yielded negative results [45]. In Ghana and Ivory coast, *M. ulcerans* has been isolated from fish, amphibians (tadpoles and adult frogs) and fecal samples mice and grasscutter [46,47].

### *M. ulcerans* survival in the environment

The optimum temperature for most *Mycobacteria* spp. is 30–32°C [48]; *M. ulcerans* grows best at 29–33°C, partly accounting for its strict extracellular nature [49], initiating infections on external body parts, its transmission and pathogenesis. Rapid intracellular replication of *M. ulcerans* has been observed in amphibian cell lines at 28°C [49]. The survival of *M. ulcerans* in the environment is temperature specific [49]. Also, availability of appropriate nutrients is essential for its growth and survival. *M. ulcerans* has a broader spectrum of growth requirements including carbohydrates, alcohols, amino acids, fatty and carboxylic acids that serve as carbon and energy sources [50]. Phosphorus and nitrogen containing solutes are also essential for *M. ulcerans* nucleic acid synthesis. In the laboratory, *M. ulcerans* is routinely cultured on media supplemented with ‘Oleic Albumin Dextrose Catalase’ and glycerol. Oleic Albumin Dextrose Catalase contains oleic acid, bovine albumin, sodium chloride, dextrose and catalase. The long-chain fatty acids are essential for its metabolic activities, and glycerol serves as a carbon source. In hostile environments such as nutrient limitations and extreme temperature, bacteria engage adaptive survival mechanisms [51]. For example, *Vibrio cholerae* exist in a viable, nonculturable state upon nutrient deprivation [52]. Nutrient deprivation in *Escherichia coli* has no effect on cell viability and integrity, however it induces a temperature dependent reduction in cell-culturability [51]. In *S. aureus*, multiple-nutrient (glucose, amino acid and phosphate) starvation results in a loss of viability, decrease in cell size and increased resistance to acid shock and oxidative stress [53]. For *Mycobacterium* species, *M. smegmatis* has been shown to undergo a cellular differentiation to form small resting cell morphotype when exposed to mild nutrient starvation conditions (phosphate buffered saline with fatty acids that can be metabolized by mycobacteria) [54]. Starved *M. tuberculosis* cells showed altered morphology and staining properties [55]. It is unclear what happens when *M. ulcerans* is nutrient deprived, however its growth dynamics is similar to typical growth kinetics of lag to log and finally to death phase [56]. Unlike normal bacteria cells, *M. ulcerans* takes a relatively longer period to grow in culture with a doubling time of about 3.5 days [57]. Fast growing *M. smegmatis* shows visible colonies on Middlebrook 7H10 agar plates in less than 7 days, *M. ulcerans* will take about 4–8 weeks.

### *M. ulcerans* stress response mechanisms

**Figure 2.**
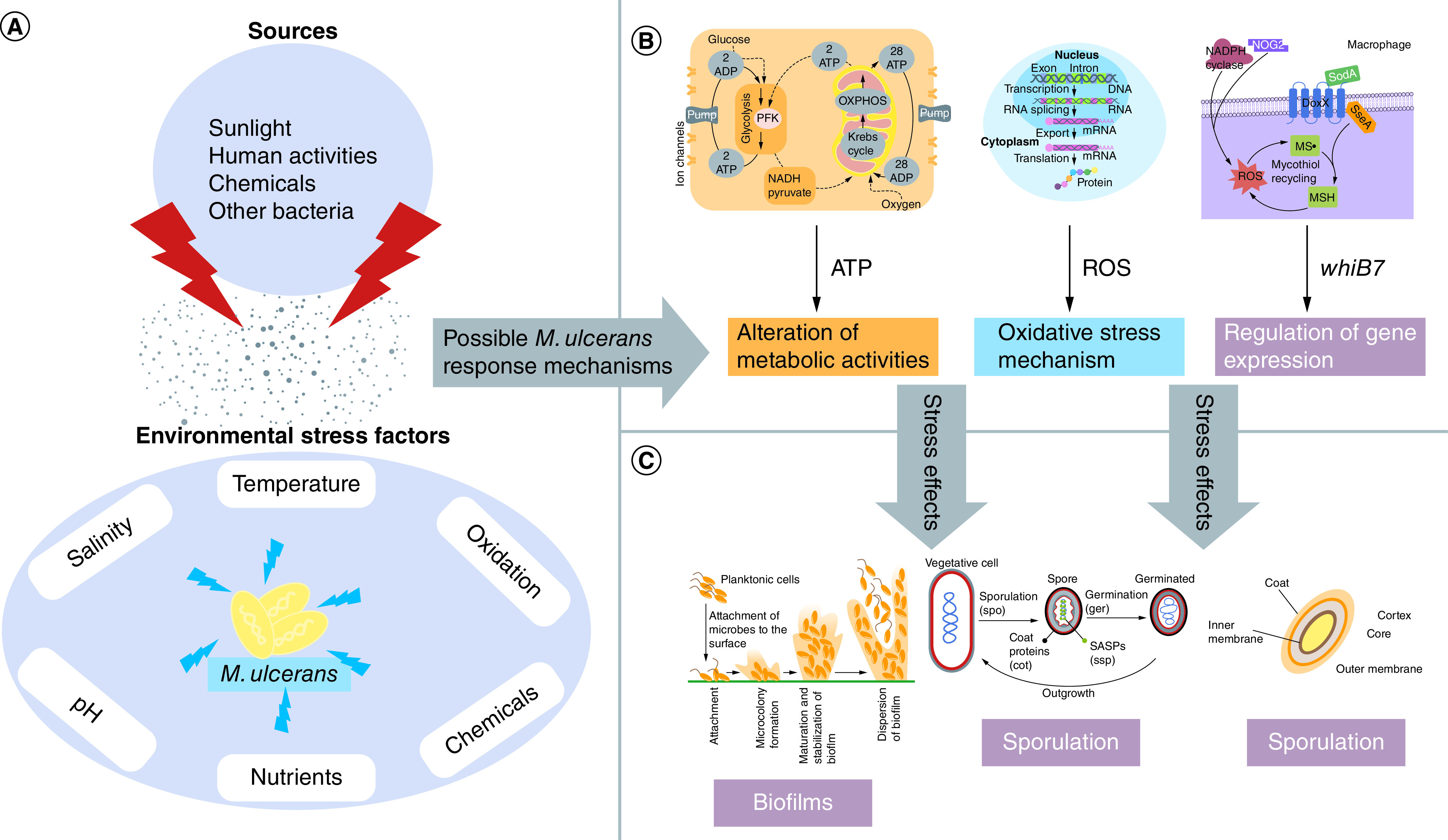
*M. ulcerans* response mechanisms to external stress factors. **(A)** Environmental stress factors that affect the survival of *M. ulcerans* and their sources. **(B)** Possible stress response mechanisms that are mounted by mycobacterium in response to environmental stress. **(C)** Stress response mechanisms or an alteration of cellular processes under stress conditions that may result in dormancy or the formation of distinct cell types such as endospores or cellular aggregates such as biofilms.

Mycobacteria species are exposed to environmental stresses including low pH, high temperature, nutrient depletion, oxidation and salinity [58]. Since these conditions are not mostly favourable, *M. ulcerans* have developed stress response mechanisms by altering their metabolic activities [59]. This is achieved by expressing specific genes and subsequent synthesis of proteins to survive this hostile environment. Also, expression of certain essential genes might be halted; for example, *sigE, sigH, sigB* control oxidative stress responses and are required for vegetative growth [60]. This response mechanism initiates morphological changes which might include decrease in cell size or rate of cell division and resistance to stress factors or agents, thereby increasing virulence or pathogenicity [61]. This might also make *M. ulcerans* to be dormant or exist in a latent state with the cells nonreplicating, however viable (Figure 2) [62].

*M. ulcerans* also engage a ‘stringent response’ mechanism against nutritional stress [62]. This involves the accumulation of nucleotide tetraphosphate (ppGpp) in the cell to downregulate the expression of ribosomal proteins and biosynthetic enzymes [62]. These proteins also activate the expression of other genes that are involved in resistance to stressed conditions. This stringent response mechanism is also common in *M. tuberculosis, M. smegmatis* and *M. leprae* [55]. Formation of specialized cells including spores and multicellular aggregates such as biofilms are potential response mechanisms [63]. Species of mycobacteria can form biofilms [40] and endospores [63].

### Sporulation as a stress response mechanism

Bacterial cells can differentiate into specialized cells called spores in response to starvation and stress [64]. Endospores are the toughest of these cells, the longest surviving and remain dormant in the environment for many years [65]; this persistence contributes to the geographic distributions of spores in ecosystems [66]. Endospores can withstand high temperatures, toxic/acidic solvents, oxidizing agents (superoxide and hydrogen peroxides), irradiation (UV) and antibiotics. Endospore formation plays a critical role in bacterial resistance to antimicrobial agents. Spores are resistant to antibiotics and disinfectants that will otherwise readily kill vegetative forms of the same cell. Bacteria endospores may encounter these extreme conditions either in the external (natural) environment or experiments that mimic natural conditions in the laboratory [67]. Bacterial endospores produced by species of the *Bacillus* and *Clostridium* are the most resistant cellular structures [68]. Sporulation in *B. subtilis* has been used as a model system to improve the understanding of various basic processes in bacteria including mycobacteria [69]. Spore-like particles with structural similarity to *Bacillus* spp. endospores have been identified in *M. marinum* [63]. These particles were identical to well-known spores in terms of their physical, biochemical, morphological and cell biological properties, while maintaining the genetic materials which identify them as *M. marinum* [63]. For instance, the presence of dipicolinic acid, a major biochemical feature of bacterial endospores [70]. Transmission electron microscopy showed that the spore-like particles are morphologically similar to spores of *Bacillus* spp. [63]. *M. bovis* bacillus Calmette–Guerin also forms particles similar to *M. marinum* spores under similar conditions; an indication that sporulation is not restricted to *M. marinum*. It is, however, likely a general characteristic or an adaptation strategy among mycobacteria [63]. Since *M. ulcerans* evolved from *M. marinum* and 98% genetically similar [25], they likely shared similar sporulation profiles.

### Model of endospores formation in *M. ulcerans*

**Figure 3.**
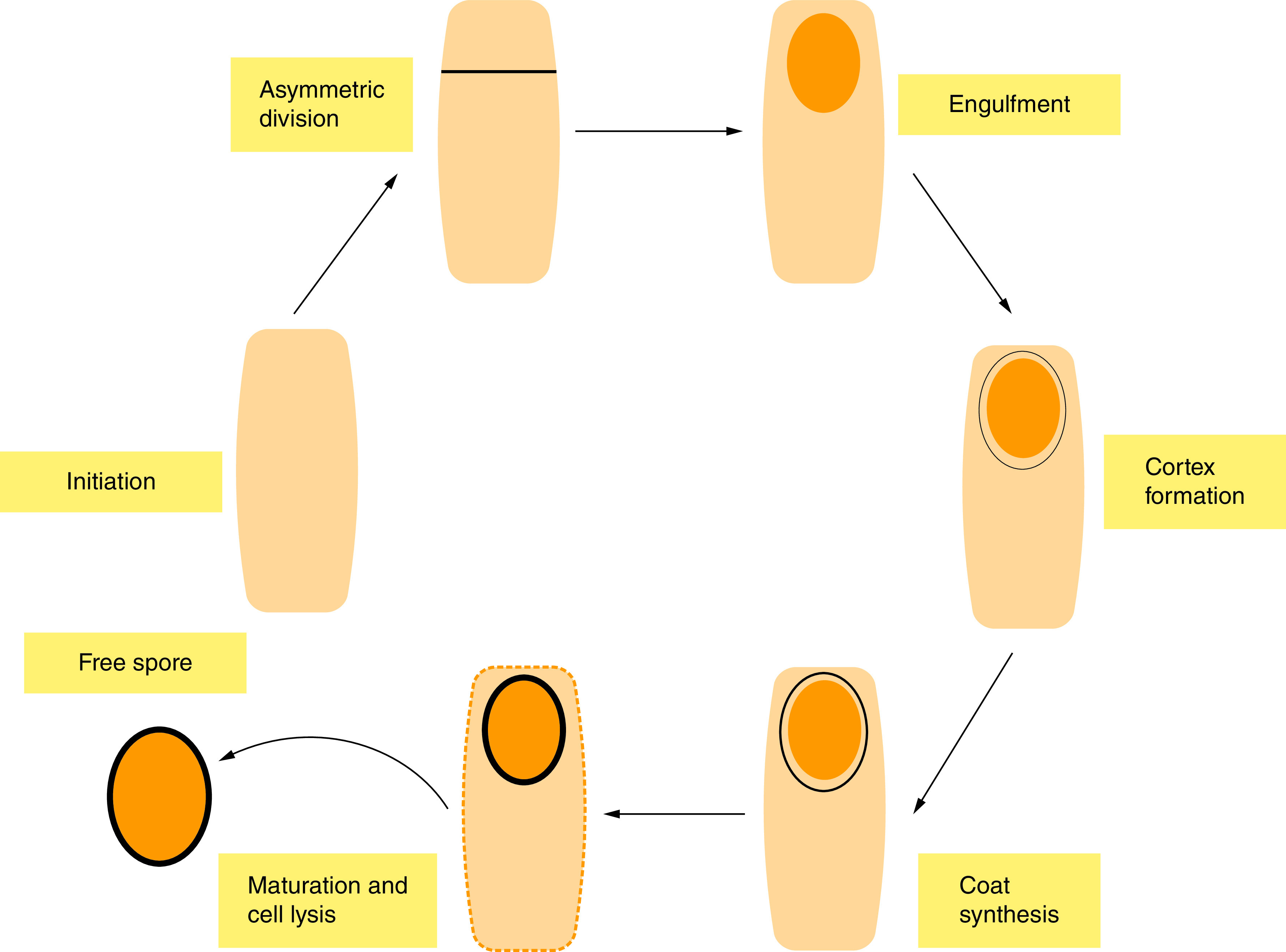
A schematic-proposed overview of the sporulation process in *M. ulcerans*. The diagram illustrates only key morphological stages of the cycle; The cell differentiates into a smaller prespore, which goes through several stages, accompanied by differential gene regulation to form a mature spore that is highly resistant to stress.

As *M. ulcerans* encounters unfavorable environmental conditions, it switches cellular processes in response [69]. Such a process includes its ability to produce endospores, although the development of spores may not be immediate; however, it is initiated as its growth rate and metabolic pathway becomes altered. This alteration enables *M. ulcerans* to explore other alternative processes before committing to sporulation activities, which is similar to the prespore formation process in *B. subtilis* [69]. These alternative responses might include activation of sliding motile structure as found in *M. smegmatis* [71] to pursue new nutrient sources, to release antibiotics that can kill other competing microbes and produce hydrolytic enzymes that can degrade extracellular proteins. This cascade of activities could further trigger the sporulation process in *M. ulcerans* especially when nutrients such as dextrose, oleic acid, glycerol and in some cases, nitrogen are in short supply. Sporulation is initiated by the phosphorylation and activation of the *spo0A*, a master regulator transcription factor [72], which induces asymmetric division and triggers subsequent transcription of other sporulation markers such as *spoIIE* and the *Spo* loci. The *Spo loci* and *spoIIE* encodes spore-developmental regulators and proteins that form a normal polar septum respectively [69]. The cells would thereby differentiate into a smaller prespore, later developing into a spore and relatively larger mother cell that is also required for spore formation (Figure 3).

This would be complemented with different gene expression profiles influenced by sporulation-specific RNA polymerase, Sigma factors: δ^F^ in the prespore and δ^E^ in the mother cell. The prespore is engulfed by the mother cell about after cell division. A significant change in transcription should occur after completion of the engulfment stage, leading to the activation of δ^G^ in the prespore and δ^K^ in the mother cell [73]. The change in gene regulation should be accompanied by specific morphogenesis, which leads to the development of the resilience observed in matured spores [73].

### Sporulation associated genes in mycobacteria

Comparative genomics showed that mycobacterial genome has putative gene orthologs similar to sporulation markers in *B. subtilis* and *Streptomyces coelicolor* [63]. Some of these genes, for example *sigF, sigJ, spo0A, spoVE* when expressed are involved in the sporulation process [69]. For example, while *sigJ* is a key regulator for desiccation tolerance, *spoVE* encodes for proteins that are involved in spore cortex and peptidoglycan synthesis. This expression is controlled by master regulons (transcriptional co-regulated operons), protein profiling of spore contents and RNA sequence profiling of sporulation gene expression [74]. These genes are characterized based on the sporulation cycle (Table 1), rather than their biochemical functions [74].

**Table 1.** Sporulation associated markers.

Strain	Sporulation markers	Detection method	Ref.
*Mycobacterium marinum*	*spoVK, cotSA, yrbC, spoVE, Soj, spoIIIE, spo0A, spoIIAB, sigA, sigF*	Dot-blot hybridization	[68]
*M. avium* Subsp. *Paratuberculosis*	*MAP 122 (kinB^+^)*, *MAP 0260 (kinA^+^)*, *phoR (kinC^+^)*, *MAP 1002c (spo0A)*, *MAP 3780 (spo0B)*	PCR	[30]
*M. tuberculosis*	*sigF*	PCR	[79]
Other spore forming environmental pathogens			
*Bacillus* spp.	*cotA, crbB, cotS, sspA, sspE, sspA, sotF, coxA, cspD, Hbs, phoA, sleB, spsK, spoVK, sigF, sigA, spoIIIE, sotsA, spsK, spo0A, Soj, sotD*	Proteomics analysis (SDS-PAGE, Mass Spectrometry), Transcriptional Profiling	[71,73]
*Clostridium* spp.	*spoVS, spoIIE, sigF, spo0A, ftsAZ, aad, ctfa, ctfb, spoIIR, spoIIQ, spoIIIG, spoIIAA, spoIIAB, spoIIGA, spoIIIJ*	DNA microarray	[74,75]
*Streptomyces coelicolor*	*whiG, whiL, whiA, whiB, parAB, ftsZ, hupS*	Transcriptional analysis	[76,77,78]

Despite the clinical, industrial and environmental importance of spores, sporulation mechanisms are mostly restricted to *B. subtilis* and *B. anthracis.* There has been relatively little information on sporulation in *Clostridium* spp. [75]. Although comparative genome analyses have profiled certain genes associated with the regulation of sporulation process, there is paucity of data on the essential set of sporulation genes required for producing a complete and viable heat-resistant spore [76]. Some of these essential genes may be triggered by expression of mutation in the parental cells [76]. In addition, some sporulation genes may be essential only in certain species or mutants due to the occurrence of several alternative regulatory pathways [76]. Therefore, defining essential genes that are required for sporulation is nontrivial, however needed to profile bacterial behavioural patterns especially in hostile environments.

## Conclusion

This review provided succinct insights into the evolution and genomic profiles of *M. ulcerans* as the causative agent of BU. It highlighted possible transmission patterns, associated transmission factors and survival strategy of *M. ulcerans* as an environmental pathogen. Also, the mechanisms of spore formation in response to stress conditions as driven by genetic factors including sporulation markers, which might influence the strain pathogenesis and BU severity.

## Future perspective

*M. ulcerans* is evolving with increasing threat to public health, especially in developing countries including Africa. Its involvement in BU is challenging as the transmission patterns and profiles are unclear. Evidence of its genome diversity pointed to its ability to develop stress response and survival mechanisms in hostile environments, an indication that *in vivo* it has the tendency to surmount the odds of the immune systems thereby increasing the severity of its associated infections. The expression of associated genetic factors that triggers virulence of the strains apart from ‘mycolactone’ would provide insights into emerging phenotypic traits such as sporulation and consequently influence disease dynamics. It is obvious that the paucity of data on the pathogenesis, reservoirs, evolution and genomic signatures of *M. ulcerans* provide progressive and notable reasons for further studies. Robust profiling techniques with sensitive assays, genomic algorithms and proteomic tools would provide clarity and balanced perspective into the immunology that can facilitate hypothesis driven research.

**Executive summary**

**Background**

- *M. ulcerans* is an environmental pathogen that evolved from *M. marinum* and they share similar genetic features.
- As informed and facilitated by their genetic similarities, it is likely they employ similar mechanisms for survival and disease transmission.

**Buruli ulcer**

- The genomic profiles and diversity of *M. ulcerans* (the causative agent of Buruli ulcer) might confer survival advantage especially in hostile environment and against immune factors.
- Sporulation is a phenotypic response mechanism of *M. ulcerans* to unfavorable conditions and expression of spore-forming markers might trigger the development of pathogenic and virulent traits.

**Future perspective**

- The expression of associated genetic factors that triggers virulence of *M. ulcerans* aside mycolactone would provide insights into emerging phenotypic traits including sporulation and consequently influence disease dynamics.
